# Possible alleviation of symptoms and side effects through clinicians’ nocebo information and empathy in an experimental video vignette study

**DOI:** 10.1038/s41598-022-19729-w

**Published:** 2022-09-27

**Authors:** M. C. Meijers, J. Stouthard, A. W. M. Evers, E. Das, H. J. Drooger, S. J. A. J. Jansen, A. L. Francke, N. Plum, E. van der Wall, Y. Nestoriuc, E. Dusseldorp, L. M. van Vliet

**Affiliations:** 1 Health, Medical and Neuropsychology Unit, Department of Health-, Medical and Neuropsychology, Institute of Psychology, Leiden University, Wassenaarseweg 52, 2333 AK Leiden, The Netherlands; 2 Department of Medical Oncology, Netherlands Cancer Institute, Amsterdam, The Netherlands; 3 Medical Delta, Leiden University, TU Delft, Erasmus University Rotterdam, Delft, The Netherlands; 4 Centre for Language Studies, Radboud University Nijmegen, Nijmegen, The Netherlands; 5 NIVEL, Netherlands Institute of Health Services Research, Utrecht, The Netherlands; 6 Department of Medical Oncology, University Medical Center Utrecht, Utrecht University, Utrecht, The Netherlands; 7 Department of Clinical Psychology, Helmut-Schmidt-University/University of the Federal Armed Forces, Hamburg, Germany; 8 Systemic Neuroscience, University Medical Centre Hamburg-Eppendorf, Hamburg, Germany; 9 Methodology and Statistics Unit, Institute of Psychology, Leiden University, Leiden, The Netherlands

**Keywords:** Cancer, Cognitive neuroscience, Social neuroscience, Quality of life

## Abstract

To alleviate anti-cancer treatment burden in advanced breast cancer, patient-clinician communication strategies based on nocebo-effect mechanisms are promising. We assessed distinct/combined effects on psychological outcomes (e.g. anxiety; main outcome) and side-effect expectations of (1) nocebo information about the (non)pharmacological origin of side effects, and (2) clinician-expressed empathy through reassurance of continuing support. Furthermore, we explored whether information and empathy effects on side-effect expectations were mediated by decreased anxiety. In a two-by-two experimental video-vignette design, 160 cancer patients/survivors and healthy women watched one of four videos differing in level of nocebo information (±) and empathy (±). Regression and mediation analysis were used to determine effects of information/empathy and explore anxiety’s mediating role. Anxiety was not influenced by empathy or information (Stai-state: p = 0.281; p = 0.410, VAS p = 0.387; p = 0.838). Information improved (specific) side-effect coping expectations (p < 0.01). Empathy improved side-effect intensity expectations (p < 0.01 = specific; p < 0.05 = non-specific/partial) and specific side-effect probability expectations (p < 0.01), and increased satisfaction, trust, and self-efficacy (p < 0.001). No mediating effects were found of anxiety on expectations. Mainly empathy, but also nocebo information improved psychological outcomes and—mainly specific—side-effect expectations. Exploring the power of these communication elements in clinical practice is essential to diminish the anti-cancer treatment burden in advanced breast cancer.

## Introduction

While anti-cancer treatment in advanced illness, such as incurable breast cancer, may prolong patients’ *quantity* of life, it may also be associated with impaired *quality* of life. Many patients experience considerable psychological and physical symptoms and side effects, with 30–74% experiencing symptoms such as anxiety, nausea, insomnia, and fatigue^1^. Treatment side effects are, apart from disease progression, key factors in the decision to stop treatment^2^ and may contribute to poorer overall quality of life^3^.

In a quest to find better ways to alleviate burdensome symptoms and side effects, we may need to look beyond merely pharmacological treatments and focus on communication strategies based on nocebo-effect mechanisms. In the context of side effects, nocebo effects are those side effects that are not solely attributable to pharmacological substances of the anti-cancer treatment^4,5^ but are due to, or exacerbated by, patient expectations, past experiences^6^, or the therapeutic context^7^. It is thought that nocebo effects play a greater role in the non-specific (non-dose-dependent) side effects of treatments, such as insomnia^4^, although they can also aggravate specific (dose-dependent) side effects, such as hair loss^8^.

Indeed, there is abundant evidence that manipulating patients’ expectations of treatments is a mechanism that elicits nocebo effects^9,10^. Merely informing patients about possible treatment side effects can increase the numbers who report those side effects and can intensify worry and concern^4,11–13^. In contrast, intriguing new studies (including open-label placebo studies^14^) seem to indicate that informing patients about the existence and functioning of the nocebo effect can actually reduce side effects^15–17^. These studies indicate that educating patients about the non-pharmacological origin of side effects may strengthen their perceived control over, primarily non-specific effects^15,17^, and (for patients with advanced cancer) may even decrease side-effect occurrence^16^ and severity (Michnevich et al., submitted). In these studies, psycho-education occurred outside the medical consultation; it is not yet known whether the same results would be found if clinician-expressed information about the nocebo effect were integrated into the doctor-patient consultation.

Another mechanism potentially capable of alleviating nocebo effects is clinician-expressed empathy. There is evidence that clinician-expressed empathy can reduce pain^18^ and improve patient satisfaction and quality of life^19–23^. In advanced cancer, a particularly powerful empathic behaviour is to reassure patients that they will receive continuing support and will be well taken care of: this has been found—mainly in experimental studies—to reduce feelings of anxiety and increase self-efficacy and information recall^7,20,24,25^. However, it has not yet been determined whether clinician-expressed reassurance of continuing support may lessen—expected—side effects.

Moreover, it is important to explore the *pathways* through which clinician-expressed nocebo-effect information and empathy may alleviate the—expected—side effects of anti-cancer treatment. In general, clinician-expressed empathy is often thought to alleviate patient anxiety^20,21^. Furthermore, anxiety sensitivity has also been found to positively correlate with nocebo responses^26,27^ and has been associated with patients’ physical impairment^28^. Therefore it might be promising to examine whether the potential effects of clinician-expressed information and empathy on expected specific and/or non-specific side effects might be routed via a decrease in patients’ anxiety.

Against this background, the aims of this experimental study conducted in the setting of advanced breast cancer are twofold. First, we investigate how nocebo information and clinician-expressed empathy may affect patients’ psychological outcomes (e.g., anxiety; main outcome) and expectations regarding side effects. Specifically, we will examine the distinct and combined effects of (1) nocebo information about the non-pharmacological origin of side effects, and (2) clinician-expressed empathy through reassurance of continuing support. Second, we explore whether the effect of information and empathy on side-effect expectations may be mediated by a decrease in participants’ anxiety. We will include expectations regarding occurrence, intensity, and coping in relation to both specific (dose-dependent) and non-specific (non-dose-dependent) side effects. Pursuing these aims can provide insight into the causal effect of communication strategies in the setting of advanced cancer and may pave the way towards alleviating patients’ psychological symptoms and side effects.

## Methods

### Design

An experimental video-vignette study was employed, with a 2 × 2 design. Four role-played video vignettes were developed, depicting a consultation between an oncologist and a patient with advanced breast cancer, in which potential treatments (chemotherapy) were discussed. All video vignettes were equal in communication and content; the only variations concerned the level of nocebo information (with (+) or without (−) a nocebo explanation) and empathy (with (+) or without (−) added reassurance of continuing support) (see Table 1 for the design). Reporting conformed to CONSORT guidelines^29^.

**Table 1:** Overview of the content of the four videos (‘conditions’).

Video 1: Nocebo information−/Empathy−	Video 2: Nocebo information−/Empathy+
Video 3: Nocebo information+/Empathy−	Video 4: Nocebo information+/Empathy+

### Script and video development

The videos were developed following the steps postulated by Hillen et al.^30^. The scripts were created by the research group (researchers, clinicians, and patient representatives). Content and manipulations were based on clinical observations^25^, a qualitative preparatory study^31^, previous experimental video-vignette^20,21,25,32,33^ and open-label studies^15,34^, and research/clinical/patient expertise of the research group. To ensure the internal (i.e., manipulation success) and external (e.g., realism) validity of the videos, an expert group of clinicians, researchers, patients/survivors, and healthy women were involved in creating/piloting the videos (see Online Appendix 1 for the development procedures, including internal/external validity evaluations). The final scripts were role-played by professional actors. The exact manipulations are displayed in Table 2; the total final scripts are displayed in Online Appendix 2.

**Table 2:** Nocebo information and empathy manipulations as used in the video vignettes.

Nocebo-information manipulation (nocebo explanation)	Empathy manipulations (reassurance of continuing support)
**Oncologist:** What not everyone knows is that side effects are not only caused by the medication itself. If people expect a side effect, have previously had a troubling side effect, or are anxious about that happening—all these things can make side effects worse. This has been shown by scientific research, so it’s not at all unusual for this to happen. **Patient:** Like you sometimes get a headache as soon as you read the information leaflet about certain medication? **Oncologist:** Yes, exactly—that’s a good example. And it doesn’t make the headache any less real or not as bad. Negative experiences, expectations, and anxieties can worsen physical reactions and side effects, such as headaches. Maybe knowing this will help make sure you suffer less from these side effects in the future. Or that you can cope better with them. And maybe this will be because you succeed in paying less attention to those side effects or because you are less anxious about them **Patient:** Ok. That’s good to know.	(Reassurance 1) **Oncologist:** I want you to know we will really look out for you, support and guide you throughout the chemotherapy process. And by ‘we’ I mean myself but also the entire team of breast-cancer nurses and doctors. (Reassurance 2) **Oncologist:** And please do know, whether it’s better or worse than anticipated, that you are not alone. We will take good care of you, the best possible care. (Reassurance 3) **Oncologist:** And once again: when you do start chemotherapy, if you run into any issues at all, you can always call us. Within or outside office hours.

### Ethics

The study was approved by the Ethics Committee of Leiden University, Department of Psychology [2021-01-27-L.M. van Vliet-V1-2909], and was registered at the Dutch Trial Registration (NTR NL8992, on 21/10/2020). All methods were performed in accordance with relevant guidelines and regulations, and all participants signed informed consent at the time of participation.

### Participants and sample size

Adult (18 > older) female cancer patients/survivors and healthy women with sufficient command of Dutch could participate. All participants (cancer patients, cancer survivors and healthy women) acted as Analogue Patients (APs) when viewing the video, putting themselves in the shoes of the patient with advanced breast cancer in the video. The validity of the AP methodology has been shown^35,36^, and previous studies found no differences in response between healthy women and cancer patients^20,33^. We included only female participants as it might be more difficult for male participants to identify with the female actor-patient.

In line with previous studies^20,21^ we used anxiety as a primary outcome. Based on a previous study^21^ using a similar experimental design that found a medium effect size (Cohen’s d = 0.28) for the primary outcome (i.e. anxiety), it was estimated^20^ that a sample size of 144 was required to attain 80% power for detecting two main effects and one interaction effect at p < 0.05. To obtain valid responses of 144 participants, we aimed to recruit up to 160 participants.

### Recruitment and procedures

Participants were recruited online via patient organizations (e.g., Dutch Breast Cancer Society (BVN)) or social media (e.g. LinkedIn, Twitter), and through advertisements in shops, requests to former participants in our studies on cancer communication who consented to be contacted again, personal contacts, and snowballing procedures. Particular efforts were made to recruit women from non-western migrant backgrounds (e.g., via patient organization Mammarosa and key contact persons), as they are often underrepresented in research^37^. After reading an online advertisement text, participants could access a webpage (hosted via Qualtrics) containing the information letter and electronic consent form. After providing informed consent, they entered the experiment webpage. First, background characteristics were assessed. Next, Qualtrics stratified participants into (1) current cancer patients and (2) cancer survivors and healthy women, and assigned them equally and randomly to one of the four videos. We monitored inclusion to ensure both groups were sufficiently represented. Next, final outcome measures were assessed, and at study end participants were debriefed and could receive $5.90 (5 euro) reimbursement. All data were collected anonymously. Participants could contact the research team for questions and emotional support and could leave the study at any time without consequences.

For non-western participants recruited via patient organizations, participation was supported by the research team in person or via video conference (Zoom.us). If necessary, patients were assisted with completing the questionnaires.

### Measures

Questionnaires were composed in collaboration with patient representatives. The following measures were assessed.

#### Pre-video

##### Demographics

Sociodemographic and cancer-related characteristics using self-created questionnaires.

##### Personality characteristics

(1) Trait anxiety (STAI-trait)^38^ (2) Optimism (LOT-R^39^), (3) Coping styles monitoring (attending to threatening information) and blunting (avoiding threatening information) (TMSI shortened version^40^); (4) Coping information needs (Degree to which patients want to be informed about (a) proposed medical treatment, (b) potential side effects of proposed treatment (0–10 Numerical Rating Scale (NRS) ‘no information at all’ to ‘as much information as possible’), adapted from a previous study^15^);

##### Psychological outcome

Anxiety: (a) State anxiety (STAI-state)^SPS:refid::bib3838^ (main outcome), (b) current anxiety levels (0–100 Visual Analogue Scale (VAS), ‘not at all’ to ‘very much’^22,41^).

##### Anticipated side effects

(a) General side-effect expectations, (b) general side-effect experiences^16,42^, (c) current side effects (0–100 VAS ‘not at all ‘to ‘very much’), all adapted from previous studies^16,43^.

#### Post-video

##### Psychological outcomes

i. Anxiety: (a) State anxiety (STAI-state)^38^ (main outcome), (b) current anxiety levels (0–100 VAS, ‘not at all’ to ‘very much’^22,41^); The post–pre video anxiety difference scores were used for all analysis.
ii. Satisfaction with the communication in the consultation (0–10 NRS ‘not satisfied at all’ to ‘extremely satisfied’), adapted from previous study^21,22^;
iii. Trust in the doctor (0–10 NRS ‘no trust at all’ to ‘full trust’, self-created);
iv. self-efficacy (i.e. the feeling of being able to handle the future) (0–10 NRS ‘very little’ to ‘very great’)^20^;

##### Side-effect (coping) expectations

For a list of 13 pre-defined side effects (determined in collaboration with the oncologists involved and divided into the groups ‘specific’ (i.e., hair loss, nausea, diarrhea, impaired immune system functioning, neuropathy), ‘non-specific’ (i.e., loss of interest/apathy, insomnia, being abrupt/irritability) and ‘partially specific/non-specific’ (i.e. cognitive impairment, fatigue, headache, concentration problems, rash), we assessed participants’ expectations regarding: (a) probability: probable occurrence of side effects (0–10 NRS scale, ‘not probable at all’ to ‘very probable’, self-created question adapted from^16,43^, (b) intensity: intensity (severity) of side effects (0–10 NRS scale, ‘not at all intense’ to ‘very intense’ using a modified version of the GASE^44^, adapted from^15^), (c) Coping: coping with side effects (0–10 NRS scale, ‘not handling at all’ ‘handling very well’, using a modified version of the GASE^44^ adapted from^15^).

##### Manipulation success

Participants assessed the extent to which the oncologist a. provided information about the non-pharmacological origin of side effects, and b. provided reassurance of continuing support (0–10 NRS scales ‘not at all’ to ‘completely’).

### Analysis

First, background characteristics were described. One-way ANOVA and χ^2^ tests were performed to compare the background characteristics between the four conditions. Variables that differed between groups were included as covariates (i.e., control variables) in subsequent analysis. Second, manipulation successes were determined with independent t-tests. Third, to assess whether data from current cancer patients and cancer survivors/healthy women could be pooled, we compared their responses to the four conditions on the anxiety and side-effect outcomes. Fourth, using regression analysis, the influence of the following pre-video characteristics on anxiety and side-effect outcomes was assessed (as they might influence communication preferences): age^45^, education^45^, trait anxiety^46^, optimism^47^, (in)direct chemotherapy experiences^48^, migrant background^49^, coping^20,50^, treatment and side-effect information need, general side-effect expectations^51^ and side-effect experiences, current side effects. The three characteristics (number chosen due to power limits) with the strongest effects (p < 0.01) were used as control variables in, fifth, hierarchical regression analysis to assess the main and interaction effects of ‘information’ and ‘empathy’ (using contrast coding, − 0.5 and 0.5) on all outcome measures. Interaction effects were eliminated from the model if insignificant. We applied an appropriate transformation for negatively skewed data (the log10 of the inverse). For independent variables, migration background was transformed into two dummy variables (with native Dutch as reference group). To ease interpretation, the raw (uncontrolled) mean scores and Cohen’s d were described for nocebo information and empathy. Lastly, using PROCESS^52^ mediation analysis, we explored the effects on side-effect expectations of information and empathy via anxiety-reduction. Analyses were conducted using SPSS Version 25.0 at p < 0.01 (p < 0.05 trend significance).

## Results

### Sample

**Figure 1:**
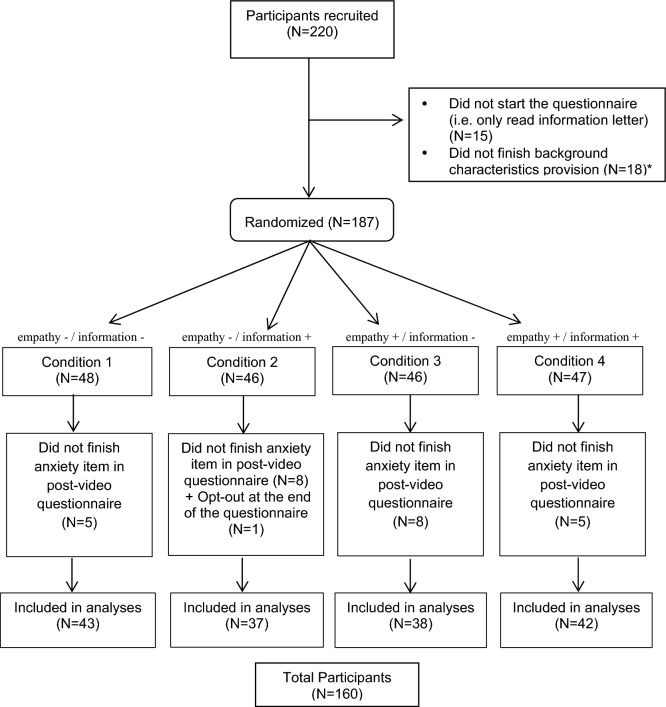
CONSORT diagram showing randomization assignment and participant flow by the group. *Randomization took place after background characteristics had been provided. Drop-out during background characteristics provision was n = 33; drop-out after randomization was n = 28. Data collection took place between February 8 and April 19, 2021.

When we reached our required sample size of 160 participants (at April 19th), 60 other participants already dropped-out due to one of the reasons as mentioned in Fig. 1 (p. 10). At the end, one participant chose to opt out (data were removed). The 160 women who completed the main outcome (STAI-state anxiety) were included in our analysis (Fig. 1). These 160 participants had a mean age of 52.93 years (SD 12.14), had mainly attended higher education (67%), and were mainly of Dutch origin (81%). There were no significant differences in background characteristics between the conditions (see Table 3).

**Table 3:** Background characteristics of participants (by condition).

Variables	Information− Empathy− N = 43	Information− Empathy+ N = 37	Information+ Empathy− N = 38	Information+ Empathy+ N = 42	Total N = 160	
	Mean (SD)	Mean (SD)	Mean (SD)	Mean (SD)	Mean (SD)	F (df); (p)
Age	51.60 (14.13)	51.12 (12.82)	53.35 (10.85)	55.52 (10.21)	52.93 (12.14)	F (3.156) = 1.10; p = 0.35
Trait anxiety (possible range: 20–80)	38.79 (9.87)	37.32 (11.16)	38.24 (8.59)	35.95 (8.60)	37.58 (9.56)	F (3.156) = .70; p = 0.55
Optimism (possible range: 0–24)	16.42 (4.04)	16.84 (4.02)	16.42 (4.65)	16.57 (4.36)	16.56 (4.23)	F (3.156) = .08; p = 0.97
Monitoring (possible range: 6–30)	21.05 (4.77)	20.14 (4.50)	20.92 (3.96)	21.07 (3.65)	20.81 (4.22)	F (3.156) = .42; p = 0.74
Blunting (possible range: 6–30)	18.91 (3.63)	18.95 (3.98)	18.34 (3.95)	20.24 (3.72)	19.13 (3.84)	F (3.156) = 1.80; p = 0.15
Treatment information need (possible range: 0–10)	9.58 (.82)	9.22 (1.13)	9.42 (.76)	9.20 (.83)	9.36 (.90)	F (3.156) = 1.76; p = 0.16
Side-effect information need (possible range: 0–10)	8.98 (1.81)	8.76 (1.59)	9.24 (.97)	8.83 (1.36)	8.95 (1.47)	F (3.156) = .79; p = 0.50
General expectations side effects (possible range: 0–100)	52.16 (26.66)	52.38 (33.64)	59.26 (27.75)	47.29 (24.81)	52.62 (28.28)	F (3.156) = 1.21; p = 0.31
General experiences side effects (possible range: 0–100)	48.70 (30.50)	41.68 (31.85)	49.00 (30.20)	46.69 (32.88)	46.62 (31.22)	F (3.156) = .44; p = 0.72
If current medication, to what extent side effects (possible range: 0–100)	36.42 (37.80)	26.87 (32.44)	30.03 (33.11)	25.62 (34.27)	29.86 (34.53)	F (3.156) = .82; p = 0.49

	N (%)	N (%)	N (%)	N (%)	N (%)	χ^2^ (df)=; (p)
Highest education*						(Fisher exact) p = .55
Low	4 (9.3)	1 (2.7)	2 (5.3)	6 (14.3)	13 (8.1)	
Intermediate	10 (23.3)	12 (32.4)	10 (26.3)	8 (19.0)	40 (25.0)	
High	29 (67.4)	24 (64.9)	26 (68.4)	28 (66.7)	107 (66.9)	
**Occupation**						(Fisher exact) p = 0.97
Paid employment	18 (41.9)	19 (51.4)	21 (55.3)	18 (42.9)	76 (47.5)	
Disabled/sick leave	15 (34.9)	10 (27.0)	9 (23.7)	15 (35.7)	49 (30.6)	
Housewife	1 (2.3)	1 (2.7)	2 (5.3)	0 (0)	4 (2.5)	
Retired	5 (11.6)	4 (10.8)	4 (10.5)	7 (16.7)	20 (12.5)	
Student	3 (7.0)	2 (5.4)	1 (2.6)	1 (2.4)	7 (4.4)	
Unemployed	1 (2.3)	1 (2.7)	1 (2.6)	1 (2.4)	4 (2.5)	
**Marital status**						χ^2^ (3) = 2.30; p = 0.52
Married (+ registered partnership)	23 (53.5)	20 (54.1)	26 (68.4)	24 (57.1)	93 (58.1)	
Unmarried (also includes cohabitant, divorced, widowed)	20 (46.5)	17 (45.9)	12 (31.6)	18 (42.9)	67 (41.9)	
**Migrant background**						(Fisher Exact) p = 0.80
Native Dutch	38 (88.4)	30 (81.1)	28 (73.7)	34 (81.0)	130 (81.3)	
Western immigrant	2 (4.7)	3 (8.1)	5 (13.2)	4 (9.5)	14 (8.8)	
Non-Western immigrant	3 (7.0)	4 (10.8)	5 (13.2)	4 (9.5)	16 (10.0)	
**Prior experience of chemotherapy**						χ^2^ (6) = 8.47; p = 0.21
Own experience	24 (55.8)	17 (45.9)	19 (50.0)	24 (57.1)	84 (52.5)	
Relative’s experience	13 (30.2)	10 (27.0)	14 (36.8)	16 (38.1)	53 (33.1)	
No experience	6 (14.0)	10 (27.0)	5 (13.2)	2 (4.8)	23 (14.4)	
**Current medications**						χ^2^ (3) = 1.45; p = 0.70
Yes	29 (67.4)	23 (62.2)	26 (68.4)	24 (57.1)	102 (63.7)	
No	14 (32.6)	14 (37.8)	12 (31.6)	18 (42.9)	58 (36.3)	
**Relation to cancer**						χ^2^ (9) = 4.92; p = 0.84
I have had cancer	16 (37.2)	9 (24.3)	15 (39.5)	14 (33.3)	54 (33.8)	
I have curable cancer	6 (14.0)	5 (13.5)	3 (7.9)	4 (9.5)	18 (11.3)	
I have incurable cancer	10 (23.3)	10 (27.0)	8 (21.1)	14 (33.3)	42 (26.3)	
I have not had cancer	11 (25.6)	13 (35.1)	12 (31.6)	10 (23.8)	46 (28.7)	

*Lower education (< secondary school); middle (secondary school + vocational education); high (higher vocational education or University).

### Manipulation success

The manipulations succeeded. In the videos with added nocebo information, the clinician was more strongly perceived as providing an explanation that side effects can have both pharmacological and non-pharmacological (psychological) origins (present: M = 8.06, SD = 1.77; absent: M = 2.05, SD = 2.05, p < 0.001). In the videos with the empathy manipulations, the clinician was perceived as providing more reassurance of continuing support than in the videos without empathy (present: M = 8.64, SD = 1.40; absent: M = 5.11, SD = 2.60, p < 0.001).

### Pooling of data

As current patients and healthy women/survivors responded the same to all but one video (information+/empathy+ for anxiety Stai-state, Online Appendix 3), data were pooled for the main analysis.

### Control variables

The three participant characteristics that had the strongest association (p- < 0.001, data not shown) with outcome measures were pre-video trait anxiety, migrant background, and treatment information needs. These variables were included as control variables in the subsequent regression model (Model 2, Table 4).

**Table 4:** Main and interaction effects of nocebo information and empathy.

Model 1—uncontrolled main effects (+ interaction effect if significant)	Nocebo information		Empathy		Nocebo information × Empathy									
	B	p	B	p	B	p								
Anxiety (Stai_state)	0.09	0.260	− 0.03	0.694										
Anxiety (VAS)	0.05	0.575	− 0.02	0.816										
Probability of specific	− 0.06	0.420	− 0.25	0.001*										
Intensity of specific	0.05	0.516	− 0.24	0.002*										
Coping of specific	0.20	0.011	0.03	0.680	0.20	0.009*								
Probability of non-specific	− 0.02	0.765	− 0.16	0.043**										
Intensity of non-specific	0.05	0.531	− 0.20	0.011**										
Coping of non-specific	0.09	0.273	0.15	0.057										
Probability of partial	0.06	0.426	− 0.17	0.032**										
Intensity of partial	0.09	0.289	− 0.19	0.016**										
Coping of partial	0.12	0.133	0.08	0.295										
Satisfaction (VAS)^	− 0.06	0.462	0.26	0.001*										
Trust^	− 0.05	0.492	0.25	0.001*										
Self-efficacy	− 0.08	0.324	0.32	< 0.001*										

Model 2—controlled main effects (+ interaction effect if significant)	Nocebo information		Empathy		Nocebo information x Empathy		Migrant background (Western migrant vs native Dutch)		Migrant background (non-Western migrant vs native Dutch)		Trait anxiety		Treatment information need	
	B	p	B	p	B	p	B	p	B	p	B	p	B	p
Anxiety (Stai_state)	0.06	0.410	− 0.8	0.281			0.10	0.165	− 0.01	0.891	− 0.47	< 0.001*	< 0.01	0.984
Anxiety (VAS)	0.02	0.838	− 0.06	0.387			0.10	0.187	− 0.04	0.604	− 0.46	< 0.001*	0.01	0.890
Probability of specific	− 0.05	0.486	− 0.21	0.006*			0.00	0.996	0.11	0.171	0.08	0.298	0.20	0.013
Intensity of specific	0.05	0.549	− 0.23	0.005*			0.04	0.631	0.16	0.043**	− 0.03	0.736	0.13	0.103
Coping of specific	0.20	0.010*	0.05	0.558	0.19	0.013**	0.05	0.482	− 0.12	0.131	− 0.14	0.077	0.15	0.049**
Probability of non-specific	− 0.02	0.787	− 0.13	0.095			0.02	0.802	0.22	0.006**	0.19	0.016**	0.08	0.277
Intensity of non-specific	0.04	0.628	− 0.19	0.013**			0.11	0.148	0.32	< 0.001*	0.14	0.072	0.02	0.791
Coping of non-specific	0.07	0.348	0.15	0.059			0.11	0.161	− 0.06	0.459	− 0.17	0.041**	0.08	0.295
Probability of partial	0.07	0.386	− 0.14	0.086			0.002	0.984	0.18	0.027**	0.07	0.364	0.17	0.028**
Intensity of partial	0.08	0.323	− 0.16	0.036**			0.11	0.156	0.22	0.005*	0.07	0.394	0.14	0.082
Coping of partial	0.12	0.135	0.09	0.257			0.05	0.508	− 0.15	0.056	− 0.19	0.019**	0.13	0.096
Satisfaction (VAS)^	− 0.04	0.618	0.30	< 0.001*			− 0.08	0.322	− 0.03	0.736	0.02	0.823	0.22	0.006*
Trust^	− 0.04	0.614	0.28	0.001*			− 0.10	0.189	− 0.01	0.987	− 0.04	0.585	0.17	0.033**
Self-efficacy	− 0.07	0.338	0.33	< 0.001*			− 0.03	0.690	0.08	0.314	− 0.04	0.619	0.08	0.326

Significant values are in [bold].

B = standardized beta *p < 0.01 **p < 0.05 (trend significance).

Transformation of these negatively skewed variables did not alter the effects, so the non-transformed variables were maintained.

### Main and interaction effects of nocebo information and empathy

#### Nocebo information

**Figure 2:**
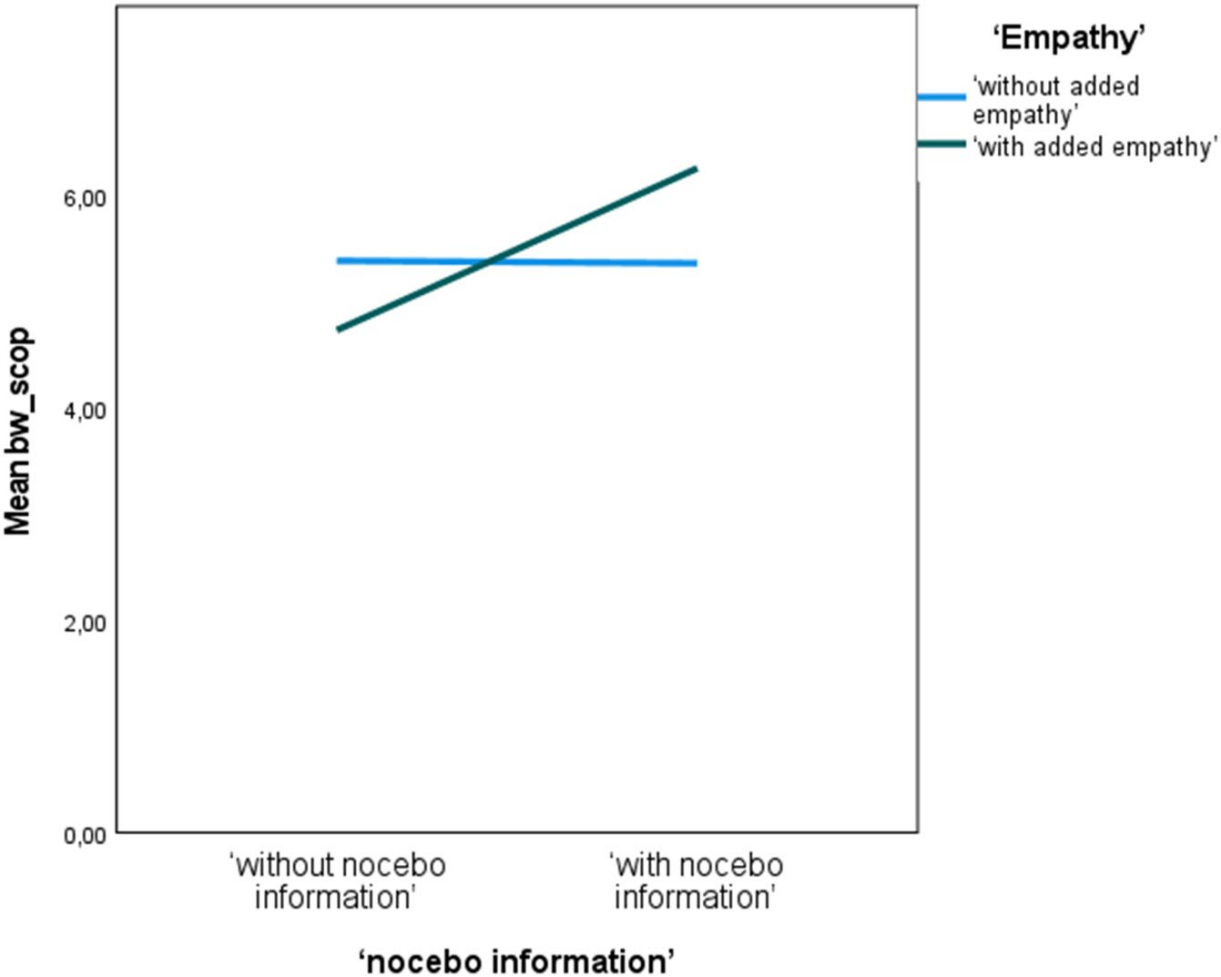
Interaction effect of nocebo information and empathy for coping expectations regarding specific side effects.

As demonstrated in Table 4, in controlled models the nocebo explanation did not influence APs’ anxiety levels (Stai-state: p = 0.410, VAS p = 0.838), or their feelings of satisfaction, trust, and self-efficacy (p > 0.05). The nocebo explanation improved coping expectations regarding specific side effects but this effect was dependent on the level of empathy (the interaction effect was significant; p < 0.01). This interaction effect revealed that when empathy was present the nocebo explanation improved coping expectations but without empathy the information decreased the expectations (see Fig. 2). Nocebo information did not influence expectations concerning the intensity or probability of side effects (p > 0.10). Outcomes for present and absent nocebo explanation are displayed in Table 5, demonstrating that apart from specific coping, for which the effect size (uncontrolled) was 0.40, all other effects were small.

**Table 5:** Raw uncontrolled mean scores and effect sizes of nocebo information condition and empathy condition on all outcomes.

	N	Nocebo information			Added empathy		
		Without (−)	With (+)	Effect size*	Without (−)	With (+)	Effect size*
		M (SD)	M (SD)	(*d*)	M (SD)	M (SD)	(*d*)
Anxiety (Stai_state) (pre-post-video difference score) (possible range: − 30 to 30)	160	14.35 (8.61)	15.80 (7.78)	0.18	15.28 (8.81)	14.86 (7.61)	− 0.05
Anxiety VAS (pre-post video difference score) (possible range: − 100 to 100)	160	49.44 (35.07)	52.40 (34.19)	0.09	51.46 (35.49)	50.37 (33.79)	− 0.031
Probability of specific (possible range: 0–10)	160	7.06 (1.62)	6.81 (1.59)	− 0.16	7.34 (1.28)	6.51 (1.79)	− 0.53
Intensity of specific (possible range: 0–10)	159	6.36 (1.88)	6.49 (1.84)	0.07	6.86 (1.66)	5.97 (1.94)	− 0.49
Coping of specific (possible range: 0–10)	159	5.08 (1.78)	5.83 (1.94)	0.40	5.37 (1.85)	5.55 (1.95)	0.09
Probability of non-specific (possible range: 0–10)	160	4.11 (2.48)	3.93 (2.71)	− 0.07	4.44 (2.66)	3.59 (2.46)	− 0.33
Intensity of non-specific (possible range: 0–10)	159	3.81 (2.49)	4.00 (2.81)	0.07	4.42 (2.67)	3.36 (2.53)	− 0.41
Coping of non-specific (possible range: 0–10)	159	4.80 (1.88)	5.20 (2.27)	0.19	4.68 (1.98)	5.33 (2.16)	0.32
Probability of partial (possible range: 0–10)	160	4.55 (2.46)	4.79 (2.40)	0.11	5.07 (2.48)	4.26 (2.30)	− 0.34
Intensity of partial (possible range: 0–10)	159	4.30 (2.38)^	4.64 (2.48)	0.14	4.92 (2.45)	4.01 (2.33)	− 0.38
Coping of partial (possible range: 0–10)	159	5.08 (1.76)	5.54 (1.93)	0.25	5.15 (1.74	5.49 (1.97)	0–18
Satisfaction VAS (possible range: 0–10)	159	7.62 (1.86)	7.48 (1.86)	0.08	7.09 (2.00)	8.03 (1.55)	0.52
Trust (possible range: 0–10)	159	7.87 (1.73)	7.75 (1.67)	− 0.07	7.40 (1.93)	8.24 (1.30)	0.52
Self-efficacy (possible range: 0–10)	159	6.52 (2.01)	6.31 (1.91)	− 0.11	5.81 (1.93)	7.04 (1.78)	0.66

*Effect sizes are positive when the condition (with vs. without) has a positive effect. The effect sizes are uncontrolled.

#### Empathy

As demonstrated in Table 4, in controlled models reassurance of continuing support did not influence anxiety levels (Stai-state: p = 0.281, VAS p = 0.387) but did increase feelings of satisfaction, trust, and self-efficacy (p < 0.001). Following reassurance, APs also expected side effects to be less intensive (p < 0.01 = specific; p < 0.05 = non-specific and partial), and specific side effects less probable to occur (p < 0.01; non-specific and partial probability were p > 0.05); however, their coping expectations did not improve (p > 0.05). APs’ outcomes for with and without added reassurance are displayed in Table 5, demonstrating that all the significant effects of empathy represent a medium effect size (ranging from d = 0.49 for specific intensity to d = 0.66 for self-efficacy).

#### Control variables

While most associations between control variables and outcomes were insignificant, it did emerge that after the videos, APs with higher trait anxiety were less anxious than those with lower trait anxiety; and those with higher information needs were more satisfied than those with lower information needs. APs with a non-western background tended to expect somewhat worse side-effect outcomes (Table 4).

### Mediating role of anxiety

In no case was the effect of nocebo information and empathy on side-effect expectations mediated by APs’ state anxiety levels (for direct, indirect, and total effects see online Appendix 4). The direct effects of information and empathy on side effects were in line with the results of the regression analyses.

## Discussion

In this online experimental video-vignette study—among Aps—we explored how nocebo information and clinician-expressed empathy affect patients’ general psychological well-being and expectations of experiencing side effects. Specifically, we aimed to determine how psychological outcomes (e.g., anxiety, main outcome) and side-effect expectations were affected by the clinician providing nocebo information about the non-pharmacological origin of side effects, and/or expressing empathy through reassurance of continuing support: we examined the distinct and combined effects of these two variables. In addition, we explored whether the effect of information and empathy on APs’ side-effect expectations might be mediated by a decrease in their anxiety.

Results indicated that neither information nor empathy had an effect on APs’ anxiety levels; only empathy improved other psychological outcomes. Empathy also led APs to expect that specific side effects were less probable and would be less intense. Information improved APs’ expectations of being able to cope with specific side effects. No mediating role of anxiety was found.

In our study, the nocebo explanation had little influence overall on APs’ expected side-effect occurrence and intensity. This finding contradicts previous studies in which nocebo explanations led to actual symptom reduction among patients with weekly headaches^16^, and to increased feelings of perceived control^17^ and decreased severity of side effects among cancer patients (Michnevich et al., under review). This contradiction might be due to our experimental design incorporating the nocebo explanation into the oncologist-patient encounter, as opposed to longer psycho-education conducted in clinical practice^15–17^; however, it also brings to light some peculiarities. First, our finding that nocebo information improved (only) APs’ coping expectations regarding *specific* side effects contradicts the thinking of the previous studies that nocebo information affects the experience of more *non-specific* side effects of treatments^15,17^ (Michnevich et al., under review). Given our results, we can only speculate that, when it came to coping expectations, the specific side effects may have been more readily known to our APs, since the term ‘side effects’ usually refers to pharmacological effects^16^. Second, we should note that although the clinical study of Michnevich et al. did find an effect on side-effect *experiences*, it too failed to find an effect on side-effect *expectations*, which raises the question whether expectations even are the pathway via which nocebo explanations influence actual side effects. Clinical follow-up studies are needed to better understand the pathway(s) of how a nocebo explanation may affect experiences of side effects (whether specific or non-specific).

The finding that information did not influence APs’ psychological outcomes (anxiety (main outcome); satisfaction with the communication; trust in the doctor; self-efficacy), may seem less surprising, as cognitive and affective communication serve different patient needs^48,53^. At the same time, it should not be overlooked that a clinician explaining the non-pharmacological origin of side effects did *not increase* psychological distress. Especially since it can be argued that the majority of the nocebo expectations in (particularly cancer) patients comes from the necessities for transparency about the options and consequences of treatment. While clinicians are legally and morally imperative to convey side-effect information, this exact information might also harm. Qualitative comments from our pilot studies and consultations with patients showed that there is a fine line between the realization that side effects have a non-pharmacological component and the idea that side effects are all in one’s head, which our intervention successfully seemed to balance.

Unlike previous studies^21,32,42,48^, in the present research empathy did not affect patient anxiety, and although it is acknowledged that empathy is no magic bullet^54^, this finding remains difficult to explain. While we could argue that discussion of a relatively young mother’s incurable cancer may evoke anxiety independently of communication style, this is not in line with previous video-vignette studies^20,21^. That being said, empathy did, in line with other studies, influence psychological outcomes such as trust^33,55^ and satisfaction^20,23^, providing further evidence that short empathic statements can influence psychological outcomes for the better^32,56^.

At the same time, also somewhat unexpectedly, empathy led to APs’ expecting specific side effects (as opposed to non-specific, as discussed above) to be less intensive and less probable to occur. This finding raises questions about the active ingredients in current nocebo-explanation interventions, in which information provision is often combined with an empathic attitude^15–17^. Indeed, the few studies that have disentangled clinician-provided positive information from an empathic attitude have found that it is only when positive clinician-information is provided in a warm and caring manner that psychological outcomes such as anxiety, satisfaction, and stress are improved^21,57,58^. These studies’ suggestion that physician empathy is of utmost importance for patients, and essential for information provision, is in line with our notable effect sizes for the enhanced empathy condition. By comparison, clinical studies have found similar small to moderate effect sizes of patients’ response expectancies on overall cancer treatment-related side effects (r = 0.153–0.431)^59,60^. This adds to the evidence that empathy may decrease patients’ physical experiences and symptoms^19,23,61^, and may also facilitate information provision in advanced cancer settings^31,32^. As such we may assume that integrating empathy into the doctor-patient consultation could be a—clinically relevant, factor for alleviating the burden of anti-cancer treatment.

While it is important—in the interests of increasing the evidence base of communication—to disentangle the pathways via which various communication elements operate, our study did not find that anxiety plays a mediating role between information and empathy and side-effect expectations. Although anxiety has been associated with nocebo effects^26,27^, there are also previous studies which failed to find that clinician-expressed empathy improves patient outcomes via a decrease in anxiety^32,56,62^. Given the effect sizes in our study for trust (d = 0.52), self-efficacy (d = 0.66) and satisfaction (d = 0.52), future studies might consider investigating the potential mediating roles of these outcome measures.

Lastly, our study suggests that some people may be more prone to experience side effects than others depending on their personality traits (e.g., neuroticism)^63,64^, and (although this is relatively underexplored) that patients from non-western groups may expect worse outcomes than those of western origin ^65,66^, showing that communication strategies are no one-size-fits-all. Our findings showed that patients with higher trait anxiety, with greater treatment information needs, and with a non-western migration background expected to experience more side effects. Interestingly, there is some indication that both anxious patients^67^ and those with high information needs^20^ may actually benefit the most from empathy. It is important to explore how patients with a non-western background may benefit from empathy, especially since non-western minority groups may receive less empathy than western patients^68,69^.

Our study has limitations. First, despite efforts to recruit patients from non-western migrant backgrounds, the majority of our participants were native Dutch, highly educated women. Given the online format, people without internet access (3% in the NL, mainly third-grade educated elderly > 65)^70^ could not participate easily. Furthermore, we did not explore demographic differences between the 60 participants who dropped out and the 160 who completed the study as stated in our rules for data handling as within the informed consent. This limits the generalizability of our results^54^. Second, caution is needed towards the significant effects found for our secondary outcome variables (Table 4) because we did not correct for multiple testing, and therefore the overall significance level was inflated. Third, scripted studies remain proxies for clinical interactions, especially as we only assessed side-effect expectations and did not measure actual experiences, so results can only with caution be generalized to clinical patients. However, video vignettes provide an ethically and methodologically sound opportunity to test causal effects of specific communication elements before follow-up studies in clinical practice. Future clinical follow-up studies with a representative sample are needed to determine the effects of nocebo explanations and reassurance of continuing support in clinical practice and should explore through which underlying mechanisms information and empathy may operate.

To conclude, as a first step we have demonstrated that empathy—and to a lesser extent nocebo information—can improve psychological outcomes and side-effect expectations in APs in an experimental setting. Exploring the power of these communication elements in clinical practice is essential to reduce the burden of anti-cancer treatment in advanced breast cancer.

### Supplementary Information

Supplementary Information.

## Data Availability

The datasets generated during and/or analysed during the current study are available from the corresponding author on reasonable request.
