# Author Correction: Possible alleviation of symptoms and side effects through clinicians’ nocebo information and empathy in an experimental video vignette study

**DOI:** 10.1038/s41598-023-42781-z

**Published:** 2023-09-29

**Authors:** M. C. Meijers, J. Stouthard, A. W. M. Evers, E. Das, H. J. Drooger, S. J. A. J. Jansen, A. L. Francke, N. Plum, E. van der Wall, Y. Nestoriuc, E. Dusseldorp, L. M. van Vliet

**Affiliations:** 1 Health, Medical and Neuropsychology Unit, Department of Health-, Medical and Neuropsychology, Institute of Psychology, Leiden University, Wassenaarseweg 52, 2333 AK Leiden, The Netherlands; 2 Department of Medical Oncology, Netherlands Cancer Institute, Amsterdam, The Netherlands; 3 Medical Delta, Leiden University, TU Delft, Erasmus University Rotterdam, Delft, The Netherlands; 4 Centre for Language Studies, Radboud University Nijmegen, Nijmegen, The Netherlands; 5 NIVEL, Netherlands Institute of Health Services Research, Utrecht, The Netherlands; 6 Department of Medical Oncology, University Medical Center Utrecht, Utrecht University, Utrecht, The Netherlands; 7 Department of Clinical Psychology, Helmut-Schmidt-University/University of the Federal Armed Forces, Hamburg, Germany; 8 Systemic Neuroscience, University Medical Centre Hamburg-Eppendorf, Hamburg, Germany; 9 Methodology and Statistics Unit, Institute of Psychology, Leiden University, Leiden, The Netherlands

Correction to: *Scientific Reports* https://doi.org/10.1038/s41598-022-19729-w, published online 27 September 2022

The original version of this Article contained errors in the dataset, where one participant was incorrectly labelled as Western immigrant and should have been labelled as Native Dutch. Another participant was incorrectly labelled as Western immigrant and should have been labelled as Non-Western immigrant.

As a result, in the Abstract,

“Anxiety was not influenced by empathy or information (Stai-state: p = 0.295; p = 0.390, VAS p = 0.399; p = 0.823).”

now reads:

“Anxiety was not influenced by empathy or information (Stai-state: p = 0.281; p = 0.410, VAS p = 0.387; p = 0.838).”

In addition, in the Results section, under the subheading ‘Main and interaction effects of nocebo information and empathy’, under the subheading “Nocebo information”,

“As demonstrated in Table 4, in controlled models the nocebo explanation did not influence APs’ anxiety levels (Stai-state: p = 0.390, VAS p = 0.823), or their feelings of satisfaction, trust, and self-efficacy (p > 0.05).”

now reads:

“As demonstrated in Table 4, in controlled models the nocebo explanation did not influence APs’ anxiety levels (Stai-state: p = 0.410, VAS p = 0.838), or their feelings of satisfaction, trust, and self-efficacy (p > 0.05).”

Also in the Results section, under the subheading ‘Main and interaction effects of nocebo information and empathy’, under the subheading “Empathy”,

“As demonstrated in Table 4, in controlled models reassurance of continuing support did not influence anxiety levels (Stai-state: p = 0.295, VAS p = 0.399) but did increase feelings of satisfaction, trust, and self-efficacy (p < 0.001).”

now reads:

“As demonstrated in Table 4, in controlled models reassurance of continuing support did not influence anxiety levels (Stai-state: p = 0.281, VAS p = 0.387) but did increase feelings of satisfaction, trust, and self-efficacy (p < 0.001).”

Table 3 contained errors in mean (SD) values for “Migrant background,” “Native Dutch,” “Western immigrant,” “Non-Western immigrant”.

Incorrect:

**Table:** 

Variables	Information−Empathy− N = 43	Information+Empathy+ N = 42	Total N = 160	
	Mean (SD)	Mean (SD)	Mean (SD)	F (df); (p)
Migrant background				(Fisher Exact) p = 0.76
Native Dutch	38 (88.4)	33 (78.6)	129 (80.6)	
Western immigrant	3 (7.0)	5 (11.9)	16 (10.0)	
Non-Western immigrant	2 (4.7)	4 (9.5)	15 (9.4)	

Correct:

**Table:** 

Variables	Information−Empathy− N = 43	Information+Empathy+ N = 42	Total N = 160	
	Mean (SD)	Mean (SD)	Mean (SD)	F (df); (p)
Migrant background				(Fisher Exact) p = 0.80
Native Dutch	38 (88.4)	34 (81.0)	130 (81.3)	
Western immigrant	2 (4.7)	4 (9.5)	14 (8.8)	
Non-Western immigrant	3 (7.0)	4 (9.5)	16 (10.0)	

Table 4 contained errors for the “Western migrant vs native Dutch” and “non-Western migrant vs native Dutch”. Adjustments have been made in Table 4.

The original Table 4 and accompanying legend appear below.

**Table 4:** Main and interaction effects of nocebo information and empathy.

Model 1—uncontrolled main effects (+ interaction effect if significant)	Nocebo information		Empathy		Nocebo information × Empathy									
	B	p	B	p	B	p								
Anxiety (Stai_state)	0.09	0.260	− 0.03	0.694										
Anxiety (VAS)	0.05	0.575	− 0.02	0.816										
Probability of specific	− 0.06	0.420	− 0.25	0.001*										
Intensity of specific	0.05	0.516	− 0.24	0.002*										
Coping of specific	0.20	0.011	0.03	0.680	0.20	0.009*								
Probability of non-specific	− 0.02	0.765	− 0.16	0.043**										
Intensity of non-specific	0.05	0.531	− 0.20	0.011**										
Coping of non-specific	0.09	0.273	0.15	0.057										
Probability of partial	0.06	0.426	− 0.17	0.032**										
Intensity of partial	0.09	0.289	− 0.19	0.016**										
Coping of partial	0.12	0.133	0.08	0.295										
Satisfaction (VAS)^	− 0.06	0.462	0.26	0.001*										
Trust^	− 0.05	0.492	0.25	0.001*										
Self-efficacy	− 0.08	0.324	0.32	< 0.001*										

Model 2—controlled main effects (+ interaction effect if significant)	Nocebo information		Empathy		Nocebo information x Empathy		Migrant background (Western migrant vs native Dutch)		Migrant background (non-Western migrant vs native Dutch)		Trait anxiety		Treatment information need	
	B	p	B	p	B	p	B	p	B	p	B	p	B	p
Anxiety (Stai_state)	0.06	0.390	− 0.8	0.295			0.13	0.065	− 0.06	0.385	− 0.46	< 0.001*	0.01	0.941
Anxiety (VAS)	0.02	0.823	− 0.06	0.399			0.18	0.100	− 0.06	0.379	− 0.46	< 0.001*	0.01	0.867
Probability of specific	− 0.06	0.459	− 0.22	0.006*			0.21	0.779	0.10	0.194	0.09	0.263	0.20	0.012
Intensity of specific	0.04	0.591	− 0.23	0.005*			0.08	0.325	0.14	0.075	− 0.02	0.828	0.13	0.100
Coping of specific	0.20	0.008*	0.05	0.537	0.19	0.016**	0.03	0.667	− 0.13	0.109	− 0.14	0.065	0.16	0.044**
Probability of non-specific	− 0.02	0.760	− 0.13	0.092			0.03	0.729	0.19	0.015**	0.20	0.011**	0.09	0.256
Intensity of non-specific	0.03	0.669	− 0.19	0.012**			0.11	0.158	0.30	< 0.001*	0.15	0.054	0.03	0.715
Coping of non-specific	0.08	0.329	0.15	0.059			0.08	0.291	− 0.05	0.521	− 0.18	0.031**	0.09	0.276
Probability of partial	0.07	0.404	− 0.14	0.082			− 0.003	0.968	0.17	0.035**	0.08	0.319	0.18	0.025**
Intensity of partial	0.07	0.339	− 0.17	0.033**			0.10	0.203	0.21	0.007*	0.07	0.370	0.14	0.069
Coping of partial	0.12	0.122	0.09	0.248			0.03	0.722	− 0.15	0.068	− 0.20	0.013**	0.13	0.093
Satisfaction (VAS)^	− 0.04	0.612	0.30	< 0.001*			− 0.07	0.365	− 0.02	0.781	0.02	0.807	0.21	0.007*
Trust^	− 0.04	0.604	0.28	0.001*			− 0.10	0.203	0.01	0.950	− 0.04	0.606	0.17	0.036**
Self-efficacy	− 0.07	0.336	0.33	< 0.001*			− 0.04	0.642	0.07	0.387	− 0.03	0.667	0.08	0.316

Significant values are in [bold].

B = standardized beta *p < 0.01 **p < 0.05 (trend significance).

Transformation of these negatively skewed variables did not alter the effects, so the non-transformed variables were maintained.

The original Article has been corrected.

