# Supplementary material for: Possible alleviation of symptoms and side effects through clinicians’ nocebo information and empathy in an experimental video vignette study
Source: Sci Rep. 2022 Sep 27;12:16112. doi: 10.1038/s41598-022-19729-w (PMC9515213; doi:10.1038/s41598-022-19729-w)
Supplement: Supplementary file 1 — Supplementary Information. [file 41598_2022_19729_MOESM1_ESM.docx]

**Possible alleviation of symptoms and side effects through clinicians’ nocebo information and empathy in an experimental video vignette study**

Maartje Meijers, Jacqueline Stouthard, Andrea Evers, Enny Das, Elrike Drooger, Sophie Jansen, Anneke Francke, Elsken van der Wal^6^, Yvonne Nestoriuc, Elise Dusseldorp, Liesbeth van Vliet

**Appendix 1 Script and video development**

**Appendix 2 Scripts** **of the four video vignettes**

**Appendix 3 Data pooling**

**Appendix 4 Mediation analysis**

**Appendix 1 – Script and video development**

Videos were developed using the 5-phase framework^1^: (1) deciding if video vignettes are appropriate, (2) developing a valid script, (3) designing valid manipulations, (4) converting the scripted consultations to video, and (5) administering the videos.

**Phase 1: Deciding if video vignettes are appropriate**

Using scripted video vignettes in an experimental design makes it possible to manipulate and standardize communication, which allows causal conclusions about the manipulated communication.

**Phases 2 and 3: Developing a valid script and designing valid manipulations**

The scripts were created by the research group (consisting of researchers, clinicians and patient representatives). The content and manipulations were based on clinical observations^2^, a qualitative preparatory study (in press), previous experimental video-vignette^2-6^ and open-label studies^7,8^, and research/clinical/patient expertise of the research group.

The content of the consultation consisted of the discussion of a chemotherapy treatment and its side effects. To increase the empathic involvement of analogue patients, a priming video was used, in which the video patient introduced herself and shared her feelings about the upcoming consultation^9,10^. Next, there was an overall introduction scene (explaining the treatment options), which was the same for all consultations. The only difference between the four scripts was the subsequent manipulated communication (nocebo information +/-, empathy +/-). The videos differed in length due to the added manipulation fragments. In line with van Vliet et al.^11^, we did not compensate for duration differences, as a) clinical consultations with various levels of empathy also differ in length^12^, and b) entering ‘filler’ communication might unintentionally influence perceptions.

To assess the internal validity (manipulation success) and external validity (realism) of the scripts, they were piloted using an online questionnaire format (i.e., Qualtrics) among an expert group consisting of clinicians, researchers, patients, survivors, and healthy women (total N=15, participation differed per validation step).

*Validation step 1 (written scripts):* For the written scripts, the expert group (clinicians (n=2), patients/survivors (n=4), researchers (n=3)) correctly identified the function of both the nocebo explanation (M=8.11, SD=1.17, possible range 0–10) and the 3 added reassurance fragments (M=8.56, SD=.73; M=8.11, SD=1.36; M=8.33, SD=1.22, possible range 0–10; see Table 1). Moreover, they perceived the scripts as partly or completely realistic (see Table 2).

The expert group provided additional comments, for example regarding the priming (‘explain that the husband did not come to the appointment because their child is sick, last minute.’), the nocebo information manipulation (‘explain/stress that side-effects are real, but that some might have a psychological component.’) and the empathy manipulation (‘make concrete who you mean by ‘we’). The scripts were adjusted on the basis of these quantitative and qualitative results.

**Phase 4: Converting the scripted consultations to video**

*Phase 4a: Preliminary videos*

Actors role-played the different scripts, and the research team filmed the priming scene, the introduction scene, the nocebo information + / empathy + script, and the nocebo information - / empathy - script.

*Validation step 2 (preliminary videos):* To assess the internal validity (manipulation success) and external validity (realism) of the videos, they too were piloted. The preliminary videos were piloted among 24 participants in total, again including the expert group – consisting of clinicians (n=2), patients/survivors(n=4), healthy women (n=1), and researchers (n=8) – as well as a group of students (n=9). Both the nocebo information manipulation (low M=0.96, SD=1.52; high M =8.5, SD=1.18) and the empathy manipulation (low M=2.71, SD=2.16; high M=8.67, SD=1.27) were successful (see Table 1). Moreover, all respondents perceived the scripts as partly or completely realistic (see Table 2). All but one of the respondents either partly (N=13, 54%) or completely (N=10, 42%) empathized with the patient in the video.

Again, the pilot participants provided overall comments and feedback about the priming (e.g., ‘It is very realistic: you can see the patient’s anguish and tension. However, the way she speaks is in my opinion still a bit ‘theatrical’. I think it could be a bit less polished.’), the nocebo information manipulation (e.g.’, the explanation could be a bit clearer’), and the empathy manipulations (e.g., ‘it was a bit too much to say ‘I’m here for you’ multiple times). The scripts were adjusted on the basis of these quantitative and qualitative results.

*Phase 4b: Final videos* Lastly, the priming and introduction scene and all four scripts were filmed by a professional video production company twice (prefinal and final version).

*Validation step 3 (prefinal videos):*

The prefinal videos were piloted among the expert group (N=8), consisting of clinicians (n=2), patient (n=2), healthy women (n=1), and researchers (n=3). Again, both the nocebo information manipulation (low M=1.37, SD =1.58; high M=7.88, SD=1.64) and the empathy manipulation (low M=3.06, SD=2.35; high M=8.13, SD=1.53) were correctly identified. Moreover, almost all respondents perceived the scripts as partly or completely realistic (see Table 1), and most respondents empathized with the patient, either partly (N=3, 38%) or completely (N=5, 63%).

Again, overall comments and feedback were provided about the priming scene (e.g., ‘I think the way the actress pauses between words is sometimes a bit artificial’) and the overall scripts (e.g., ‘the transition is a bit abrupt from the empathy manipulation to the patient clarifying whether the only options are either chemo or no treatment’). Based on these quantitative and qualitative results, some last editorial changes were made to the scripts and videos (but not to the manipulations), and the scripts were filmed.

**Phase 5: Administering the video**

The final videos were used in the experiment (see Table 3 for the time duration of the final videos and manipulations, and Appendix 2 for the scripts used).

**Supplementary Table S1.** Manipulation success across the validation steps (internal validity)

| Step | N | Nocebo information | | Empathy | |
| --- | --- | --- | --- | --- | --- |
|  |  | Without (-) | With (+) | Without (-) | With (+) |
|  |  | *M* (SD) | *M* (SD) | *M* (SD) | *M* (SD) |
| Validation step 1:  written scripts | 9 | n.a. | 8.11 (1.17) | n.a. | Fragment 1: 8.56 (.73) |
|  |  |  |  |  | Fragment 2: 8.11 (1.36) |
|  |  |  |  |  | Fragment 3: 8.33 (1.22) |
| Validation step 2:  self-recorded videos | 24 | 0.96 (1.52) | 8.5 (1.18) | 2.71 (2.16) | 8.67 (1.27) |
| Validation step 3:  prefinal, professionally recorded videos | 8 | 1.37 (1.58) | 7.88 (1.64) | 3.06 (2.35) | 8.13 (1.53) |

*Note.* Manipulation success was measured with two purpose-designed items that asked participants to rate the degree to which the oncologist a) provided information that side effects could originate both from pharmacological and psychological mechanisms (range 0-10); and b) reassured patients that they would be supported throughout the chemotherapy (range 0-10).

**Supplementary Table S2.** Realism of the scripts/videos across the validation steps (external validity)

|  | N | Priming | Introduction | Video | | | |
| --- | --- | --- | --- | --- | --- | --- | --- |
| Realism |  |  |  | Nocebo information - / Empathy - | Nocebo information - / Empathy + | Nocebo information + / Empathy - | Nocebo information + / Empathy + |
|  |  | N (%) | N (%) | N (%) | N (%) | N (%) | N (%) |
| Validation step 1: written scripts | 9 |  |  |  |  |  |  |
| Not at all |  | NA | NA | - | - | - | - |
| Partly |  | NA | NA | 5 (56) | 7 (78) | 5 (56) | 5 (56) |
| Completely |  | NA | NA | 4 (44) | 2 (22) | 4 (44) | 4 (44) |
| Validation step 2: self-recorded  videos | 24 |  |  |  |  |  |  |
| Not at all |  | - | - | 1 (4) | NA | NA | - |
| Partly |  | 10 (42) | 14 (58) | 17 (71) | NA | NA | 12 (50) |
| Completely |  | 14 (58) | 10 (42) | 6 (25) | NA | NA | 12 (50) |
| Validation step 3: prefinal  recorded videos | 8 |  |  |  |  |  |  |
| Not at all |  | - | - | - | - | - | 1 (13) |
| Partly |  | 2 (25) | 1 (13) | 3 (38) | 3 (38) | 2 (25) | 2 (25) |
| Completely |  | 6 (75) | 7 (88)* | 5 (63)* | 5 (63)* | 6 (75) | 5 (63)* |

* due to rounding, adds up to 101

**Supplementary Table S3.** Final length of the videos

|  | Priming |  | Video* | | | |
| --- | --- | --- | --- | --- | --- | --- |
|  | *(prior to every video)* |  | Nocebo information - / Empathy - | Nocebo information - / Empathy + | Nocebo information + / Empathy - | Nocebo information + / Empathy + |
| Time duration (minutes.seconds) | 1.00 | + | 6.16 | 6.44 | 7.15 | 7.43 |

* The exact length of the manipulations can be calculated on the basis of these time durations. Empathy manipulation = (6.44-6.16) 0.28 seconds. Nocebo-explanation manipulation = (7.15-6.16) 59 seconds.

**Appendix 2 - Scripts of the four video vignettes**

**Abbreviations:**

**Camera perspectives:**

**CAM2= focus on the patient**

**CAM3= focus on the oncologist (over the shoulder)**

**CAM1= complete focus (on both oncologist and patient)**

**P=patient**

**O=oncologist**

**Scene: Priming**

| **Verbal communication** | **Non-verbal and camera instructions** |
| --- | --- |
| P: I’m Elly de Jong. I’m a mom with 3 kids. They are the most important thing in my life. A few weeks ago, I was diagnosed with breast cancer. For a while, I had noticed a lump in my breast that seemed to be growing. My GP/Family practitioner referred me to the hospital. At the end of a day of tests in the hospital I heard it was breast cancer. Follow-up tests revealed that I have metastatic cancer. This means my disease is incurable. The last few weeks have felt like a bad movie; I had breast cancer?! It still feels like it’s someone else it’s happening to. I’m here today to talk about possible treatment options. I hope there are still lots of possibilities. I’m here on my own today, because our daughter woke up feeling ill this morning and my husband had to stay with her. That’s just the way it is.  O: Mrs de Jong? | P is sitting in a waiting room.  She looks anxious and nervous. She stares a bit or looks around her restlessly.  Voice breaks.  Sigh |

**Scene: Introduction**

| **Verbal communication** | **Non-verbal and camera instructions** |
| --- | --- |
| O: Mrs de Jong. Good afternoon, please take a seat.  O: Are you on your own today?    P: Yes, our daughter woke up feeling ill this morning and we couldn’t find anyone to stay with her at such short notice. We didn’t want to reschedule this appointment, so..  O: Oh, I’m sorry to hear that. Uh, would you maybe like to record the conversation on your phone so you can replay it together later on?  P: Yes, that would be really helpful (takes out her phone).    O: So how are you doing?  P: Well… it all feels a bit surreal. As if it is not really about me. My husband and I have talked about it a lot. About the diagnosis, but also about this conversation.  O: Yes, I can imagine… it will all take a while to sink in (pause). I just want to go over the past few days and weeks with you. You came here because your GP/Family Practitioner suspected you might have breast cancer. And of course, sadly, that was indeed the diagnosis. Follow-up tests revealed that your cancer is hormone-insensitive and has spread to your lymph nodes and bones. These metastases unfortunately mean it is no longer possible to remove the cancer completely and cure you.  P: Yes, I understand that… | CAM1/T O opens the door. P sits down opposite O, who also sits down.  CAM 2: P is waiting for what is to come  CAM3: O looks engaged. He leans forward slightly, hands slightly folded. He makes eye contact. This is his standard attitude throughout all the scenes (unless otherwise instructed).  CAM 2: P looks a bit embarrassed  CAM 3: O friendly  CAM2: P looks relieved. P looks in her purse  CAM3: O looks friendly and engaged  CAM2: P looks reasonably hopeful  CAM3: O looks empathetic. O talks patiently and looks at P. He folds and unfolds his hands several times.  CAM2: P sighs, looks away a bit |
| O: Obviously it was a huge shock for you last time to hear that your illness was incurable, so we decided we would leave it till today to discuss the best possible treatment option for you and how to go on from here. Shall we discuss that now, right away, or do you have any questions you’d like to bring up first?  P: No, I’d rather discuss the options right away. I hope there’s still a lot to be done about it. I want to grasp every opportunity.  O: Well, there certainly are options. So what we’re looking at is chemotherapy. Our aim with chemotherapy would be to extend your life and preserve your quality of life.  P: Oof, so chemotherapy is the only option?  O: Yes, I’m afraid it is. Because your cancer is not hormone-sensitive, I’m afraid hormone therapy isn’t an option in your case. And neither is surgery, because the cancer has spread to your bones and lymph nodes. Immunotherapy is also not an option, because in your situation we want to make sure that we rapidly reduce the metastasis of the cancer. So, yes, that only leaves us with chemotherapy.  P: Oh ok.. Well.. Only chemo.. right… But that makes you feel really sick, doesn’t it, as well as lose your hair? | CAM3: O is calm and measured.  CAM2: P looks and sounds combative, crosses legs  CAM3: O continues speaking calmly  CAM2: P sounds a bit defeated, looks away for a bit, talks in a softer voice, sounds a bit sad.  CAM3: O looks at P calmly and seriously.  CAM2: P nods slowly and blinks her eyes, looks away and sounds anxious (‘can I handle this?’) |

**Scene: Information about side-effects, including Nocebo information manipulation**

Note: the ***bold text in italics*** is the Information manipulation. In the information + videos this information is added, in the information – videos it is omitted.

| **Verbal communication** | **Nonverbal and camera instructions** |
| --- | --- |
| O: There are different kinds of chemotherapy. The chemotherapy I have in mind *can* indeed elicit side effects. But how many and to what extent really differs per person. Hair loss and nausea are the most well-known, but some people also experience tingling in their toes and finger tips. And there are some side effects where you would need to contact us immediately, no matter what time of the day or night: like a fever, because that could indicate an infection, or diarrhea that persists longer than 24 hours.  P: Ok, that’s clear. | CAM3: Pretty neutral. Serious situation, but continues to maintain his standard attitude.  CAM2: Combative but also a bit anxious. |
| ***O: What not everyone knows is that side effects are not only caused by the medication itself. If people expect a side effect, have previously had a troubling side effect, or are anxious about that happening – all these things can make side effects worse. This has been shown by scientific research, so it’s not at all unusual for this to happen.***  ***P: Like you sometimes get a headache as soon as you read the information leaflet about certain medication?***  ***O: Yes, exactly – that’s a good example. And it doesn’t make the headache any less real or not as bad. Negative experiences, expectations, and anxieties can worsen physical reactions and side effects, such as headaches. Maybe knowing this will help make sure you suffer less from these side effects in the future. Or that you can cope better with them. And maybe this will be because you succeed in paying less attention to those side effects or because you are less anxious about them.***  ***P: Ok. That’s good to know.*** | ***CAM3: Pretty neutral. Really focused on the information. His standard attitude.***  ***CAM 2: P is looking for confirmation***  ***CAM3: Pretty neutral. Really focused on the information. Not very empathic.***  ***CAM2: P looks rather timid, a bit overwhelmed*** |
| O: You will also have a consultation with a specialist nurse, Chantal, who will tell you more about the possible side-effects and practicalities, such as how you can reach us.  P: Yes… ok. It’s hard to get my head round it. | CAM3: O is quite neutral  CAM2: P looks a bit timid, a bit overwhelmed. |

**Scene: Treatment options, including empathy manipulations**

Note: the ***bold text in italics*** are the 3 empathy manipulations. In the empathy *+* videos these segments are added, and in the empathy *–* videos they are omitted.

| **English** | **Nonverbal and camera instructions** |
| --- | --- |
| O: Yes. If you do want to start chemotherapy, we could start as soon as next week. What we’re hoping is that the chemo will slow down the tumor growth for as long as possible, though I can’t give you any guarantees that it will help. We’ll only know that once we perform a scan after a few treatment cycles. And as we said just now, you might experience side-effects. | CAM3: O responds to ‘it’s hard to get my head round it’ with understanding, nonverbally. Is quite neutral, but when he says he is not sure it will help, he is a bit more gentle in what he says and does.  CAM2: Very brief moment (this is so the next scene can be edited). |
| ***O: I want you to know we will really look out for you, support and guide you throughout the chemotherapy process. And by ‘we’ I mean myself but also the entire team of breast-cancer nurses and doctors.*** | ***CAM3: Empathetic, hands slightly looser. Attitude in line with verbal communication, but otherwise not very different from in the other scenes.***  ***Neutral*** |
| P: Ok (reflects for a moment). So if I understand it correctly, it basically boils down to either chemotherapy or nothing, but in that case I will certainly die sooner.  O: Yes, if we don’t start treatment that aims to slow down the growth of the tumor, we indeed know for certain that the cancer will continue to grow… There are some women in your situation who don’t want chemotherapy or any other treatment. In that case there’s still a lot we can do to ensure the best possible quality of life for you.  P: No, doing nothing is not an option for me. It just isn’t.  O: May I ask you what is most important in your life at the moment?  P: I have 3 children, a husband. They mean everything to me.. (silence).. I also have my job, the office. But it’s the kids, above all. For their sake I definitely want to try treatment.  O: Yes I understand, and I can completely imagine.. So shall I tell you a bit more about the chemotherapy? So you can discuss it with your husband at home and I can call you in a few days to check that you still want to start chemotherapy?  P: Yes, that’s a good idea. We have already talked about it at home, but still.  O: Okay, the chemotherapy that I would suggest consists of 3 treatment cycles. These are given every 3 weeks. After 3 cycles we will do a scan. If the chemo seems to be working – so the metastases have become smaller – and provided you’re feeling well enough, we’ll continue with another 3 cycles. The chemo is delivered via an IV, here at the daycare unit.  P: Ok. Once every three weeks. And that’s the only times I’ll come here?  O: Yes and before every session, we’ll check your blood levels and you’ll see me. If your blood levels are ok and you’re feeling well enough, you will go ahead with the next round of chemotherapy. | CAM2: Looks a bit sad, a bit questioning, rather shocked at the end  CAM3: O is thoughtful, stays calm and watches the patient’s response carefully.  CAM2: P interrupts the doctor a bit here. She again shows a fighting spirit and sounds like she knows what she wants.  CAM3: Spoken carefully  CAM2: P is a bit emotional, but not overwhelmed by emotions. More determined. Little pause in between the examples.  CAM3: O is calm and shows understanding, does not want to rush her into a decision.  CAM2: is combative and also a bit grateful.  CAM3: O is pretty empathic and calm, provides a clear explanation.  CAM2: P nods in light agreement and looks serious, as if she is now beginning to realize what she is letting herself in for.  CAM1: O nods to confirm, hands unfolded again. |
| ***O: And please do know, whether it’s better or worse than anticipated, that you are not alone. We will take good care of you, the best possible care.*** | ***CAM3: Again empathetic, hands slightly looser. Attitude in line with verbal communication, but otherwise not very different from in the other scenes.*** |
| P: Yes, um.. Well, I’ll discuss it all with my husband. Like me, he was really hoping that treatment would still be possible.  O: Right, I’ll give Chantal a call, so she can pick you up. She will also give you some written information, so you can read it all over once you get home. And I’ll call you on Thursday morning at 09.00 am – will that suit?  P: Yes, thank you. | CAM2: P sighs, but does now have some insight into what will happen next.  CAM3/M: O looks quite neutral. Is searching for something at the computer and uses both hands for that.  CAM2: P looks friendly and somewhat grateful. |
| ***O: And once again: when you do start chemotherapy, if you run into any issues at all, you can always call us. Within or outside office hours.*** | ***CAM3: Again empathetic, hands are slightly looser. Attitude in line with verbal communication, but otherwise not very different from in the other scenes.*** |

**Appendix 3 –Data pooling**

**Supplementary Table S4**: Responses to the four videos of women who currently have cancer vs. those who currently do not have cancer (healthy women or survivors)

|  | Nocebo information -  Empathy - | |  | Nocebo information -  Empathy + | | | Nocebo information +  Empathy - | | | Nocebo information +  Empathy + | | |
| --- | --- | --- | --- | --- | --- | --- | --- | --- | --- | --- | --- | --- |
|  | Currently cancer  (n=16) | Currently no cancer  (n=27) |  | Currently cancer  (n=15) | Currently no cancer  (n=22) |  | Currently cancer  (n=11) | Currently no cancer  (n=27) |  | Currently cancer  (n=18) | Currently no cancer  (n=24) |  |
|  | *M (SD)* | *M (SD)* | *p* | *M (SD)* | *M (SD)* | *p* | *M (SD)* | *M (SD)* | *p-value* | *M (SD)* | *M (SD)* | *p* |
| **Anxiety (STAI_state difference score)**  **(range: -30-30)** | 15.19 (8.47) | 14.00 (9.39) | .68 | 14.00 (4.99) | 14.41 (10.05) | .87* | 13.73 (9.59) | 17.26 (8.18) | .26 | 11.94 (6.69) | 18.00 (6.17) | **.004** |
| **Anxiety (VAS difference score)**  **(range: -100-100)** | 52.25 (34.17) | 51.74 (35.57) | .96 | 48.67 (27.78) | 45.09 (40.97) | .77 | 39.55 (38.86) | 55.56 (35.75) | .23 | 44.22 (36.37) | 60.88 (26.81) | .11* |
| **Specific probability**  **(range: 0-10)** | 7.70 (1.07) | 7.38 (1.22) | .39 | 6.21 (2.38) | 6.77 (1.54) | .39 | 6.60 (1.22) | 7.39 (1.42) | .12 | 6.44 (1.86) | 6.52 (1.62) | .89 |
| **specific intensity**  **(range: 0-10)** | 7.16 (1.88) | 6.43 (1.71) | .20 | 5.76** (2.33) | 6.07 (1.67) | .64 | 6.60 (1.59) | 7.21 (1.47) | .26 | 5.83 (2.06) | 6.11 (1.95) | .66 |
| **specific coping**  **(range: 0-10)** | 5.46 (1.52) | 5.33 (1.89) | .82 | 4.66**  (1.87) | 4.77 (1.78) | .85 | 5.35 (1.71) | 5.36 (2.12) | .98 | 6.00 (2.00) | 6.44 (1.68) | .44 |
| **nonspecific probability**  **(range: 0-10)** | 4.71 (2.50) | 4.40 (2.59) | .70 | 3.24 (2.44) | 3.91 (2.33) | .41 | 2.97 (2.01) | 4.91 (2.96) | **.05** | 3.19 (2.73) | 3.83 (2.45) | .42 |
| **nonspecific intensity**  **(range: 0-10)** | 4.47 (2.46) | 4.07 (2.60) | .62 | 3.26**  (2.35) | 3.33 (2.45) | .93 | 3.61 (2.48) | 5.06 (2.92) | .16 | 3.35 (2.86) | 3.46 (2.60) | .90 |
| **nonspecific coping**  **(range: 0-10)** | 5.00 (1.34) | 4.47 (1.77) | .31 | 4.67** (1.54) | 5.14 (2.50) | .53 | 4.57 (1.37) | 4.73 (2.65) | .86 | 5.37 (2.51) | 5.88 (1.82) | .45 |
| **partial**  **probability**  **(range: 0-10)** | 5.45 (2.54) | 4.88 (2.58) | .49 | 3.80 (2.37) | 3.98 (2.15) | .81 | 4.05 (1.60) | 5.44 (2.63) | .11 | 4.14 (2.61) | 4.89 (2.17) | .32 |
| **partial intensity**  **(range: 0-10)** | 5.15 (2.77) | 4.36 (2.27) | .32 | 3.90**  (2.42) | 3.85 (2.15) | .95 | 4.55 (1.90) | 5.48 (2.60) | .29 | 3.77 (2.65) | 4.40 (2.28) | .41 |
| **partial coping**  **(range: 0-10)** | 5.54 (1.07) | 4.87 (1.87) | .20 | 4.47** (1.79) | 5.40 (1.96) | .16 | 5.58 (1.08) | 5.01 (2.11) | .40 | 5.29 (2.39) | 6.31 (1.42) | .09 |

*Welch t-test used as the assumption of homogeneity of variances was violated (as assessed by Levene’s test for equality of variances, p<.01)

**n=14 instead of 15 due to one drop-out

**Appendix 4 – Mediation analysis**

**Supplementary Table S5**: Direct, indirect and total effect of expressed empathy or nocebo-information on expected side effects

|  | **Direct¹** | | | **Indirect**² |  | **Indirect pathways³** | | |  |  |  | **Total**⁴ | | | **Variance of the Model**⁵ |  |  |
| --- | --- | --- | --- | --- | --- | --- | --- | --- | --- | --- | --- | --- | --- | --- | --- | --- | --- |
| **Empathy** | **Β (c’1)** | **p** | **95% CI** | **B** | **95% CI (ab)** | **Β (a)** | **p** | **95% CI** | **Β (b)** | **p** | **95% CI** | **B (c)** | **p** | **95% CI** | **R2** | **F** | **p** |
| Specific probability | -.82 | **.001*** | -1.31, -.33 | -.005 | -.07, .03 | -.42 | .746 | -3.00, 2.15 | .01 | .457 | -.02, .04 | -.82 | **.001*** | -1.31, -.34 | .26 | 11.23 (1,158) | **.001*** |
| Specific intensity | -.88 | **003*** | -1.45, -.32 | -.003 | -,07, .04 | -.48 | .717 | -3.06, 2.11 | .01 | .701 | -.03, .04 | -.89 | **.002*** | -1,45, -.32 | .24 | 9.60 (1, 157) | **.002*** |
| Specific coping | .18 | .553 | -,42, .77 | -.002 | -,05, .07 | -.48 | .717 | -3.06, 2.11 | .01 | .864 | -.03, .04 | .18 | .555 | -.42, .77 | .05 | .35 (1, 157) | .555 |
| Non-specific probability | -.84 | .041 | -1.64, -.04 | -.003 | -.09, .06 | -.42 | .746 | -3.00, 2.15 | .01 | .814 | -.04, .05 | -.84 | .040 | -1.64, -.04 | .16 | 4.31 (1, 158) | .040 |
| Non-specific intensity | -1.07 | **.012*** | -1.88, -.25 | .01 | -.10, .12 | -.48 | .717 | -3.06, 2.11 | -.02 | .432 | -.07, .03 | -1.06 | .011* | -1.87, -.24 | .20 | 6.54 (1, 157) | **.012*** |
| Non-specific coping | .67 | .044 | .02, 1.32 | -.008 | -.08, .07 | -.48 | .717 | -3.06, 2.11 | .02 | .383 | -.02, .06 | .66 | .047 | .01, 1.31 | .16 | 4.03 (1, 157) | .047 |
| Partial probability | -.80 | .037 | -1.55, -.05 | -.006 | -.10, .05 | -.42 | .746 | -3.00, 2.15 | .01 | .551 | -.03, .06 | -.81 | .035 | -1.55, -.06 | .17 | 4.52 (1, 157) | .035 |
| Partial intensity | -.91 | .019 | -1.66, -.16 | .002 | -.08,.07 | -.48 | .717 | -3.06, 2.11 | -.01 | .830 | -.05, .04 | -.91 | .018 | -1.66, -.16 | .19 | 5.70 (1, 157) | **.018*** |
| Partial coping | .35 | .241 | -.24, .93 | -.006 | -,07, .07 | -.48 | .717 | -3.06, 2.11 | .01 | .500 | -.02, .05 | .34 | .248 | -.24, .92 | .09 | 1.35 (1, 57) | .248 |
| **Nocebo-information** | **Β (c’1)** | **p** | **95% CI** | **B** | **95% CI (ab)** | **Β (a)** | **p** | **95% CI** | **Β (B)** | **p** | **95% CI** | **B (c)** | **p** | **95% CI** | **R2** | **F** | **p** |
| Specificic probability | -.27 | .286 | -.78, .23 | .02 | -.03, .12 | 1.45 | .265 | -1.11, 4.01 | .01 | .370 | -.02, .04 | -.25 | .321 | -.75, .25 | .08 | .99 (1, 158) | .321 |
| Specific intensity | .12 | .695 | -.47, .70 | .01 | -.06, .11 | 1.51 | .249 | -1.07, 4.09 | .01 | .673 | -.03,.04 | .13 | .665 | -.45, .71 | .03 | .19 (1, 157) | .665 |
| Specific coping | .75 | **.013*** | .16, 1.33 | -.002 | -.09, .08 | 1.51 | .249 | -1.07, 4.09 | -.001 | .941 | -.04, .03 | .74 | **.013*** | .16, 1.33 | .20 | 6.33 (1, 157) | **.013*** |
| Non-specific probability | -.19 | .652 | -1,00, 63 | .01 | -.08, .15 | 1.45 | .265 | -1.11, 4.01 | .01 | .746 | -.04, .06 | -.18 | .671 | -,99, .64 | .03 | .18 (1, 157) | .671 |
| Non-specific intensity | .22 | .606 | -.62, 1.06 | -.03 | -.17, .10 | 1.51 | .249 | -1.07, 4.09 | -.02 | .458 | -.07, .03 | .19 | .653 | -.64, 1.02 | .04 | .20 (1, 157) | .653 |
| Non-specific coping | .38 | .259 | -.28, 1.03 | .02 | -.07, .13 | 1.51 | .249 | -1.07, 4.09 | .01 | .485 | -.03, .05 | .40 | .230 | -.25, 1.05 | .10 | 1.45 (1, 157) | .230 |
| Partial probability | .23 | .557 | -,54, .99 | .02 | -.06, .14 | 1.45 | .265 | -1.11, 4.01 | .01 | .556 | -.03, .06 | .25 | .520 | -.51, 1.01 | .05 | .42 (1, 157) | .520 |
| Partial intensity | .35 | .366 | -.41, 1.12 | -.01 | -.11, .10 | 1.51 | .249 | -1.07, 4.09 | -.01 | .821 | -.05, .04 | .34 | .374 | -.42, 1.10 | .07 | .80 (1, 157) | .374 |
| Partial coping | .44 | .135 | -.14, 1.03 | .01 | -.08, .11 | 1.51 | .249 | -1.07, 4.08 | .01 | .615 | -.03, .04 | .46 | .122 | -.12, 1.04 | .12 | 2.42 (1, 157) | .122 |

*** p<.01**
¹ The direct effect is the effect of expressed empathy or nocebo information on expected side effects, controlled for the difference score on state anxiety
² The indirect effect is the effect of expressed empathy or nocebo information on expected side effects via the difference score of patients’ state anxiety.
³ The indirect pathways are path A and B in the model. A refers to the first pathway of the mediation model: between clinician’ expressed empathy and patient’s state-anxiety levels. B refers to the second pathway of the mediation model: between patient’s state-anxiety levels and expected side effects.
⁴ The total effect is the effect of expressed empathy or nocebo-information on expected side effects, uncontrolled for the difference score on state anxiety.
⁵ Model fit: Proportion of the variance for the dependent variable that is explained by the independent variable and the mediator variable in the regression model.

**
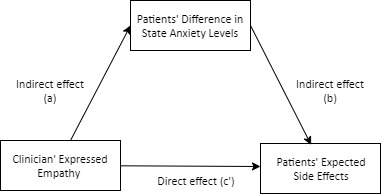
**

**Supplementary Fig 1.** The study model: The association between empathy expressed by the clinician, and patients’ expected side effects, mediated by the difference score of patients’ state anxiety levels.


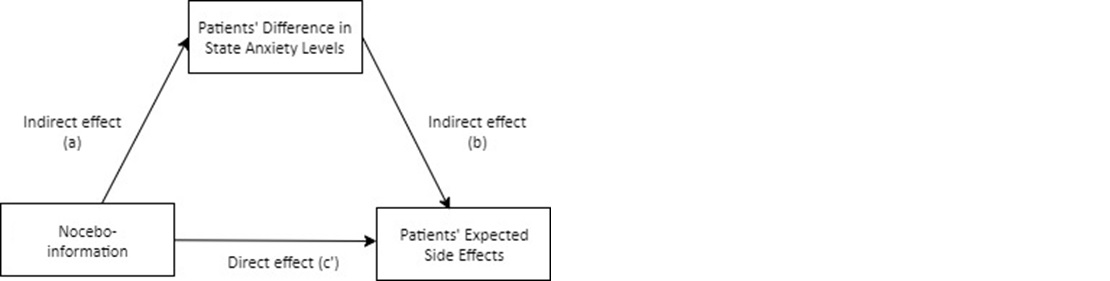


**Supplementary Fig 2.** The study model: The association between nocebo information given by the clinician, and patients’ expected side effects, mediated by the difference score of patients’ state anxiety levels.

References

1 Hillen, M. A., van Vliet, L. M., de Haes, H. C. & Smets, E. M. Developing and administering scripted video vignettes for experimental research of patient–provider communication. *Patient Educ. Couns.* **91**, 295-309 (2013).

2 Van Vliet, L. M. *et al.* The Use of Expectancy and Empathy When Communicating With Patients With Advanced Breast Cancer; an Observational Study of Clinician–Patient Consultations. *Front. Psychiatry* **10**, 464 (2019).

3 van Vliet, L. M., van der Wall, E., Plum, N. M. & Bensing, J. M. Explicit prognostic information and reassurance about nonabandonment when entering palliative breast cancer care: findings from a scripted video-vignette study. *J. Clin. Oncol.* **31**, 3242-3249 (2013).

4 van Osch, M., Sep, M., van Vliet, L. M., van Dulmen, S. & Bensing, J. M. Reducing patients’ anxiety and uncertainty, and improving recall in bad news consultations. *Health Psychol.* **33**, 1382 (2014).

5 Hillen, M. A. *et al.* All eyes on the patient: the influence of oncologists’ nonverbal communication on breast cancer patients’ trust. *Breast Cancer Res. Treat.* **153**, 161-171 (2015).

6 van Osch, M., van Dulmen, S., van Vliet, L. & Bensing, J. Specifying the effects of physician's communication on patients’ outcomes: A randomised controlled trial. *Patient Educ. Couns.* (2017).

7 Crichton, F. & Petrie, K. J. Health complaints and wind turbines: The efficacy of explaining the nocebo response to reduce symptom reporting. *Environ. Res.* **140**, 449-455 (2015).

8 Quidde, J. *et al.* Preventing adverse events of chemotherapy by educating patients about the nocebo effect (RENNO study)–study protocol of a randomized controlled trial with gastrointestinal cancer patients. *BMC Cancer* **18**, 1-8 (2018).

9 Gerrards‐Hesse, A., Spies, K. & Hesse, F. W. Experimental inductions of emotional states and their effectiveness: A review. *Br. J. Psychol.* **85**, 55-78 (1994).

10 Westermann, R., Spies, K., Stahl, G. & Hesse, F. W. Relative effectiveness and validity of mood induction procedures: A meta‐analysis. *Eur. J. Soc. Psychol.* **26**, 557-580 (1996).

11 Van Vliet, L. M., Hillen, M. A., van der Wall, E., Plum, N. & Bensing, J. M. How to create and administer scripted video-vignettes in an experimental study on disclosure of a palliative breast cancer diagnosis. *Patient Educ. Couns.* **91**, 56-64 (2013).

12 Epstein, R. M. *et al.* Patient-centered communication and diagnostic testing. *Ann. Fam. Med.* **3**, 415-421 (2005).
